# Definitions of health and social care standards used internationally: A narrative review

**DOI:** 10.1002/hpm.3573

**Published:** 2022-09-20

**Authors:** Yvonne Kelly, Niamh O’Rourke, Rachel Flynn, Josephine Hegarty, Laura O’Connor

**Affiliations:** ^1^ Health Information and Standards Directorate Health Information and Quality Authority Cork Ireland; ^2^ Catherine McAuley School of Nursing and Midwifery University College Cork Cork Ireland

**Keywords:** definition, health care, inspection, legislation, quality improvement, social care, standards

## Abstract

Setting standards is a quality improvement mechanism and an important means for shaping the provision of health and social care services. Standards comprise statements describing a process or outcome of care. Setting standards is a global practice. It would be useful to have an understanding of the underpinning definitions of standards used internationally. Therefore, the aim of this review was to examine definitions of health and social care standards used internationally and identify similarities and differences. A targeted grey literature search of standard‐setting bodies' websites and related health legislation was conducted to retrieve explicit definitions of standards. Of 15 standard‐setting bodies that were searched, 12 definitions of standards were narratively synthesised. Terms that appeared in two or more of the definitions were extracted. Counts and percentages were calculated for these terms to determine magnitude of use. The commonalities among definitions included ‘quality’ (*n* = 6, 50%), ‘statements’ (*n* = 5, 42%), ‘performance’ (*n* = 5, 42%), and ‘measureable’ (*n* = 4, 33%). The less commonly used terms were ‘processes’ (*n* = 3, 25%), ‘set’ (*n* = 3, 25%), ‘evidence based’ (*n* = 2, 17%), ‘outcome’ (*n* = 2, 17%), ‘safe’ (*n* = 2, 17%), and ‘guidance’ (*n* = 2, 17%). Explicit definitions of standards were not retrieved from health legislation documents. Standard‐setting bodies develop standards in the context of the health systems in which they are implemented; some are aspirational levels of quality, while others are minimum levels of quality. Researchers, standards developers and policy makers should be cognisant of this when comparing standards between countries.

## Key points

- In analysing 12 standards definitions, we found considerable variation.
- Some were aspirational levels of quality, others were minimum quality levels.
- Common terms were ‘quality’, ‘statements’, ‘performance’, and ‘measureable’.
- Comparing standards between countries should be conducted with caution.

## 1 INTRODUCTION

Quality improvement in health and social care is complex and achieved through many different approaches. The development and publication of health and social care standards is one approach that is adopted by many countries internationally. Standards are multi‐faceted interventions written to demonstrate the desired level of care that a service should aim to provide. Standards, therefore, aim to act as levers to improve the quality of care delivered in health and social care services.^1^ The International Organisation of Standardisation (ISO) refer to standards as a ‘formula’ that describes ‘the best way of doing something.’^2^ The Oxford English dictionary defines standards as a ‘level of quality’ or ‘level of behaviour’ or a ‘unit of measurement.’^3^ The Australian Commission on Safety and Quality in Health Care develop standards to provide quality assurance by highlighting variation in practice and ensuring that systems are in place to achieve safe and acceptable care for both service providers and for people using healthcare services.^4^ An impact report on the National Safety and Quality Health Service (NSQHS) Standards in Australia identified areas of significant improvements following implementation of their standards.^5^ Areas of improvement included: better documentation of adverse drug reactions and medication history, some hospitals reporting a 20% relative reduction in monthly cardiac arrest rates, and an increase from 36% (2010) to 98% (2015) in hospitals having antimicrobial stewardship programmes.^5^

Standards for health and social care can be used in public oversight processes such as accreditation, licensure and regulation of health and social care settings. Accreditation and licencing is a monitoring process adopted by healthcare systems to independently assess the performance and compliance of healthcare settings against pre‐determined standards.^6^ This process is used in the United States of America (USA) where, for example, some healthcare services are evaluated by the Joint Commission on Accreditation of Healthcare Organisations (JCAHO).^6^ If services are deemed compliant with standards, approval for licensure is granted. Other approaches to monitoring include inspections, where teams of experts make visits to health and/or social care settings to assess compliance with nationally endorsed standards.^7^ Such standards are used as benchmarks by monitoring bodies to measure good performances and are aspirational standards. For example, the quality standards produced by the standard‐setting body in the United Kingdom (UK), National Institute for Health and Care Excellence (NICE) can be used to determine performance levels during inspections by the regulator of health and social care services in England, the Care Quality Commission (CQC).^8^ Standards can also be used as part of a regulatory framework created through legislation. A regulatory framework can comprise of the establishment of a health and/or social care regulator and new regulations set in law that services must comply with.^7^ In addition, standards can be used to stipulate and evaluate the quality of care provided by a service. The regulator may have powers to enforce penalties should there be non‐compliance with the standards, where they are mandated. The regulators of: Australia's aged care services, Aged Care Quality and Safety Commission; New Zealand's health and disability services, HealthCERT; Ireland's health and social care services, Health Information and Quality Authority (HIQA); and Northern Ireland's health and social care services, Regulation and Quality Improvement Authority (RQIA) regulate against the relevant standards developed by their national standard‐setting bodies.^9^, ^10^, ^11^, ^12^ In contrast, the regulator of health and social care in England, CQC regulates against ‘fundamental’ standards of quality and safety.^13^ These fundamental standards have been developed by the CQC and are specifically set out in the legislation that governs the regulation.^13^ As such, they are separate to what is developed and published by NICE.

Despite the array of standards available internationally in both health and social care, there is variation in the underpinning concept of how standards are defined. The Institute of Medicine (IOM) committee on quality of healthcare in America recognised a variety in definitions for standards when describing how standards and expectations about performance can strengthen improvements in patient safety. The IOM refer to standards as ‘levels of performance.’^6^ ^(p136)^ The World Health Organisation (WHO) refer to standards as a ‘level of achievement.’^14^ ^(p9)^ Both concepts, performance and achievement have distinct defining characteristics, yet both terms have interlinked and interdependent factors; one must perform in order to achieve. This reflects a process in care versus an outcome of care and thus, applies to measuring care against practices or against the experiences and health outcomes of individuals using the services. Standard‐setting bodies such as NICE and Health Quality, Ontario have developed standards that are designed to enhance the provision of care.^8^, ^15^ Conversely, HIQA in Ireland, Standards New Zealand and the Scottish Government develop nationally endorsed standards that are structured around outcomes, with statements describing achievements in practice.^16^, ^17^, ^18^

Definitions describe the nature, scope, or meaning of a phenomenon.^19^ There is a need to identify and understand how standards are defined to enhance interpretation in the context of driving safe and quality care. It is common practice to review standards as set by other organisations internationally when a country is developing a new set of standards on a topic. Therefore, it is important to understand the difference and similarities between organisations on what they mean by the term ‘standards’ in order to inform correct interpretation. As such, the aim of this work was to examine the definitions of standards used internationally, that apply to health and social care services, and identify similarities and variations through a narrative synthesis.

## 2 METHODS

### 2.1 Search strategy

A targeted search of grey literature was undertaken in August 2020. A search strategy, comprising three stages was developed with an aim to answer the research question: how are health and social care standards defined? The initial stage included identifying standard‐setting bodies that develop and publish standards for health and social care services. Names of standard‐setting bodies were extracted from a review conducted by the Irish standard‐setting body, the Health Information and Quality Authority (HIQA), on methodologies for developing national standards and guidance for health and social care services used by organisations internationally.^20^ For the review, subject matter experts in HIQA identified standard‐setting bodies with similar responsibilities and standard development processes to themselves. This list was used as the basis for this review. Two additional standard‐setting bodies: the WHO and the National Academy of Medicine (NAM) (formally known as the IOM) were also included due to their international standard‐setting remits. The websites belonging to the standard‐setting bodies were accessed by manual searches using the Google Chrome search engine (Supplementary file 1‐List of websites and web addresses (URL)).

The second stage involved a ‘hand search’ of the relevant websites' homepages for documents pertaining to standards. The targeted website searches were conducted on 16 August 2020. Key terms used to search the websites were ‘healthcare’, ‘social care’ and ‘standards.’ The search was refined to most recent documents published on overarching standards and if this was not available, then the most recently published standards document was retrieved. The titles of the documents and table of contents were reviewed for inclusion. A document met the inclusion criteria if it had an explicit description defining standards for health and/or social care. Documents were excluded if they were not available in the English language or if a more up‐to‐date version of the standards document was retrieved. In general, the main webpage headings were screened and the search function used to search key terms. Standards documents were searched for standards definitions to confirm that they fitted the eligibility criteria. The glossary section, where available, was searched for a definition.

The third stage was a targeted search of the Health Acts or equivalent, from which each standard‐setting body was established. The relevant legislation was identified from hand‐searching the websites belonging to the standard‐setting bodies or from the standards documents retrieved in stage two. They were then accessed by manual searches using the Google Chrome search engine (Supplementary file 1‐List of websites and web addresses (URL)).

### 2.2 Search outcomes

Fifteen standard‐setting bodies were identified, 13 of which were identified from the review conducted by HIQA on methodologies for developing national standards and guidance for health and social care services used by organisations internationally.^20^ Of these 13, three standard‐setting bodies were excluded at this stage for the following reasons: NICE work closely with the Social Care Institute for Excellence (SCIE) in England^20^ and have shared quality standards, as such the SCIE, as the smaller organisation, was excluded to avoid duplication of definitions. The Swedish National Board of Health and Welfare develop National Guidelines for implementation in health, social and dental services in Sweden.^21^ This did not involve developing standards and hence standards documents were not retrieved from this website. A definition of standards could not be found in the selected standards document developed by the Australian Department of Social Services.^22^

Due to the different design and layout of individual websites, searches for relevant standards documents varied among standard‐setting bodies. Flow charts to display search methods and returns for each website are included (Supplementary file 2). The term ‘standards’ was included in the glossary for three of the identified standards documents in which case these definitions were extracted from the glossary.^4^, ^16^, ^23^ It was noted from screening these three documents that two had slightly different definitions in the glossary and the main text,^4^, ^16^ in which case, the definition used in the glossary was selected for inclusion due to its explicit nature. Seven documents were read, full text and definitions were extracted from the main text of the document.^8^, ^14^, ^15^, ^18^, ^24^, ^25^, ^26^ A chapter of a book pertaining to standards set by an organisation was read and a definition extracted from the main text.^6^ One definition was extracted from text on the organisation's webpage.^27^

Eleven Health Acts or equivalent were identified from the hand search of the standard‐setting bodies eligible for inclusion. The WHO was established by the World Health Assembly consisting of multiple member states and hence, local legislation was not identified for the WHO. The IOM was established under a congressional charter in the USA but it was not possible to access this charter. The remaining ten Acts or equivalent did not have an explicit definition explaining what is meant by ‘standard’ or ‘standards’ in the context of the legislation.^28^, ^29^, ^30^, ^31^, ^32^, ^33^, ^34^, ^35^, ^36^, ^37^ Sections with titles ‘interpretation’ or ‘definitions’ where provided,^28^, ^30^, ^33^, ^35^, ^36^ appeared to act as a glossary for some terms used in the legislation but the term ‘standard’ or ‘standards’ are not listed in these sections.^28^, ^30^, ^33^, ^36^ As such, definitions used in legislation were not eligible for inclusion in this review.

### 2.3 Data extraction

The definitions used by standard‐setting bodies were extracted and were entered into an extraction table using the following headings: standard‐setting body, origin of standard‐setting body, title of standards document and definitions. Page numbers to reference the location of the definition in the standards document where applicable, were included in the table to increase transparency of the process. The next step was extracting key terms and their frequency of use. Words and phrases that appeared in two or more definitions were retrieved and recorded.

### 2.4 Data analysis

Data abstraction was conducted and using a familiarisation technique described by Braun and Clarke (2006) as ‘reading and re‐reading,’^38^ the list of definitions and key terms were extracted. Patterns of commonalities across definitions were identified through immersion in the definition content. Counts and percentages were calculated for the key terms extracted across all the definitions to measure their magnitude of use.^39^ This was then tabulated under the headings: key terms, number of definitions that the key term was extracted from with corresponding percentages, and country of origin of standard‐setting body. The data extraction table and the table with key terms used in definitions of standards were used to narratively synthesise the included definitions. Following this, a summary of findings was created in a narrative format on the overall terminology used to define standards.

## 3 RESULTS

The final search yielded 11 standards documents and one webpage text, one from each of 12 standard‐setting bodies. 11 definitions were available from standards documents for healthcare services in Australia,^4^ USA,^6^ Ontario,^15^ Ireland,^16^ social care services in Denmark^25^ and health and social care services in England,^8^ WHO,^14^ Scotland,^18^ Wales,^23^ Northern Ireland^24^ and New Zealand (Table 1).^26^ One definition was retrieved from the standard‐setting body webpage text and was applicable to healthcare services in Denmark.^27^ A total of 10 key terms that appeared in two or more definitions were extracted and tabulated (Table 2). A key term was considered most prevalent if it appeared in five or more of the 12 definitions of standards. In addition, a key term was considered less prevalent/less frequently used if it appeared in three or less definitions.

**TABLE 1:** Definition of standards presented with details of standard‐setting bodies and relevant standards documents

Standard‐setting body^a^	Origin of standard‐ setting body	Title of standards document	Definition of Standard(s)^b^
Australian commission on safety and quality in health care	Australia	National safety and quality health service standards (2017)	‘Standard: Agreed attributes and processes designed to ensure that a product, service or method will perform consistently at a designated level.’^4^ ^(p75)^
National academy of medicine	United States of America	Setting performance standards and expectations for patient safety, to err is human (2000)	‘A minimum level of acceptable performance or results or excellent levels of performance or the range of acceptable performance or results.’^6^ ^(p132)^
National institute for health and care excellence	England	The NICE quality standards programme (2016)	‘Each standard consists of a set of specific, concise statements and related measures derived from evidence‐based guidance and produced collaboratively.’ each standard contains statements that describe priority areas for improvement.^8^ ^(p8)^
World health organization	International remit	Frequently asked questions implementing health promotion in hospitals, manual and self‐assessment forms (2006)	‘It is based on two complementary approaches of quality assessment: Standards, expressing professionally consented statements on health care structures or processes that should be in place.’^14^ ^(p7)^
Health quality Ontario	Ontario	Quality standards process and methods guide (2017)	‘Quality standards are concise sets of evidence‐based, measurable statements that provide guidance on important elements of high‐quality health care in a specific topic area.’^15^ ^(p4)^
Health information and quality authority	Ireland	National standards for safer better healthcare (2012)	‘A standard is a statement which describes the high level outcome required to contribute to quality and safety.’^16^ ^(p145)^
Scottish government	Scotland	Health and social care standards my support, my life (2017)	‘Standards are headline outcomes, and the descriptive statements which set out the standard of care a person can expect.’^18^ ^(p3)^
Welsh assembly government	Wales	Health and care standards (2015)	‘Standards are a means of describing the level of quality that health care organisations are expected to meet or to aspire to. The performance of organisations can be assessed against this level of quality.’^23^ ^(p41)^
Department of health, social services and public safety	Northern Ireland	The quality standards for health and social care (2006)	‘Standard is a level of quality against which performance can be measured. It can be described as ‘essential’‐ the absolute minimum to ensure safe and effective practice, or ‘developmental’, ‐ designed to encourage and support a move to better practice.’^24^ ^(p2)^
Danish quality model for social services	Denmark	The standard programme. Key concepts (2017)	‘A standard is a parameter for quality with specific demands and directions, which form the basis of evaluation’^25^ ^(p1)^
Standards New Zealand	New Zealand	Standards New Zealand (2017)	‘Documents that provide agreed specifications for products, processes, services, or performance.’^26^ ^(p3)^
IKAS Danish institute for quality and accreditation in healthcare	Denmark	Accreditation standards (2016)	‘Each standard includes a descriptive part, where the purpose and meaning of the standard is explained in more or less detail, as appropriate.’ ‘each standard includes a number of indicators that comprise the measurable elements of the standard set’^27^

^a^ Website Addresses (URL) for Standards‐setting bodies available in Supplementary file 1.

^b^ Text available on Standard‐setting body website.

**TABLE 2:** Key Terms used and magnitude of use in standards definitions, total *n* = 12

Key terms	N^a^(%)	Origin of Standard‐setting body
Quality	6 (50%)	WHO^14^, Ontario^15^, Ireland^16^ Wales^23^, Northern Ireland^24^ Denmark^25^
Statements	5 (42%)	England^8^, WHO^14^, Ontario^15^, Ireland^16^, Scotland^18^
Performance/perform	5 (42%)	Australia^4^, USA^6^ Wales^23^, Northern Ireland^24^, New Zealand^26^
Measure/Measurable	4 (33%)	England^8^, Ontario^15^, Northern Ireland^24^, Denmark^27^
Processes	3 (25%)	Australia^4^, WHO^14^, New Zealand^26^
Set of	3 (25%)	England^8^, Ontario^15^, Denmark^27^
Evidence‐based	2 (17%)	England^8^, Ontario^15^
Outcome	2 (17%)	Ireland^16^, Scotland^18^
Safe	2 (17%)	Ireland^16^, Northern Ireland^24^
Guidance	2 (17%)	England^8^, Ontario^15^

^a^ Number of definitions that included the key term.

### 3.1 Similarities

The most prevalent key term among the definitions was ‘quality’ (*n* = 6)^14^, ^15^, ^16^ ^,^ ^23^, ^24^, ^25^ followed by ‘statements’ (*n* = 5)^8^ ^,^ ^14^, ^15^, ^16^ ^,^ ^18^ and ‘performance/perform’ (*n* = 5).^4^, ^6^, ^23^, ^24^, ^26^ The term ‘quality’ was used in the context of: level of care,^15^ level of quality,^23^, ^24^ quality assessment,^14^ quality of a service,^16^ or used as a noun in the context of a concept of evaluation.^25^ Of the six definitions that did not include quality, it was noted that quality was in the title of three of the six standards documents that these definitions were extracted from.^4^, ^6^, ^8^ The use of ‘statements’ in the definitions may be representative of the rigorous development process that setting standards demand. Statements are defined by the Oxford English dictionary as ‘official account of facts, views or plans.’^40^ In addition, 11 of 12 organisations setting standards were established under local legislative enactment^4^, ^8^, ^15^, ^16^, ^18^, ^23^, ^24^, ^25^, ^26^, ^27^, ^41^ contributing to their official nature. ‘Performance’ was used as a noun in definitions (*n* = 4)^6^ ^,^ ^23^ ^,^ ^24^ ^,^ ^26^ and also a verb (*n* = 1),^4^ reflecting an action or process.

The term ‘measure/measurable’ featured in definitions (*n* = 4)^8^ ^,^ ^15^ ^,^ ^24^ ^,^ ^27^ and was considered in the middle between most prevalent and less frequent. ‘Measurable’ was used in the context of an adjective to describe statements^15^ or elements of the standard.^27^ In addition, ‘measure’ was referred to as a plural noun^8^ and a verb^24^ in measuring a performance.

### 3.2 Variations

The less frequent terms among the definitions included ‘processes’ (*n* = 3),^4^, ^14^, ^26^ ‘set (of)’ (*n* = 3),^8^, ^15^, ^27^ ‘evidence‐based’ (*n* = 2),^8^, ^15^ ‘outcome’ (*n* = 2),^16^, ^18^ ‘safe’ (*n* = 2),^16^, ^24^ ‘guidance’ (*n* = 2).^8^, ^15^ These key terms possess distinct defining characteristics but are interlinked with the more prevalent terms extracted from the definitions. Surprisingly, evidence‐based does not feature in more definitions, considering that all 12 standard‐setting bodies retrieved from this study undertake rigorous standard development methods underpinned by research and stakeholder engagement.^4^, ^8^, ^14^, ^15^, ^16^, ^18^, ^23^, ^24^, ^25^, ^26^, ^27^, ^31^ It is low levels of quality in the delivery of care that pose a risk to patient safety.^1^ Without a level of quality, safety does not exist and possibly explains the higher prevalence of quality in definitions over safety. Standards inform health and social care services and the public, which may explain the use of ‘guidance’ to describe standards. The inclusion of the term ‘outcome’^16^, ^18^ and ‘processes’^4^, ^14^, ^26^ in the definitions reflect the structure of the standards as being statements describing an outcome of care or describing the delivery of care. The aforementioned less frequently used terms among the definitions make up attributes that form the basis of what standards are. As such, standards are sets derived from evidence‐based practices and describe outcomes or processes, hence acting as a guidance to safe care.

Variation was evident among definitions used by two standard‐setting bodies^25^, ^27^ from the one jurisdiction. The healthcare accreditation standards developed by the Danish Institute of Quality and Accreditation describe standards as having ‘measurable elements.’^27^ The Danish Quality Model for the Social Services define a standard as a ‘parameter for quality.’^25^ The standards developed for social services in Denmark form part of a quality development process, thus highlighting contextual factors associated with defining standards.

## 4 DISCUSSION

This review extracted a list of defining attributes that described health and social care standards used by standard‐setting bodies which facilitated characterising and comparing definitions of standards. There were a variety of terms used within the extracted definitions demonstrating similarities and variations in definitions of standards used internationally.

The evolution of standards stems from the quest for patient safety and quality improvement in hospital care.^42^ Standards play an important role in quality assurance and quality improvements when used as a component of inspection practices.^43^ This may explain why ‘quality’ is the most commonly used term among the definitions. A study carried out by Reeves and Bednar in 1994 reported on quality definitions referring quality to excellence, conformance, and value and meeting customer expectations.^44^ This study compared the weaknesses and strengths of definitions used to define quality but concluded that no one definition was superior to another and quality was determined by individual situations.^44^ This perhaps explains why the WHO recommend that health systems set standards based on definitions of quality relevant at local level.^1^ The Oxford English dictionary defines quality as ‘the degree of excellence of something’ or ‘general excellence of standard or level’ or ‘a distinctive attribute or characteristic.’^45^ Synonyms and related words for quality, identified by the Oxford English Dictionary include standard, condition, value, level, and mastery.^45^ In addition, the Cambridge English dictionary lists benchmark and conformance as synonyms for quality.^46^

The IOM described standards as comparative benchmarks to which an expected level of care can be measured.^6^ Reeves and Bednar^44^ reported that quality is easier to measure when defined through conformance by a focus on efficiency and effectiveness.^44^ Quality of care, benchmark, conform, level and assurance are also reflected in the act of performance. The WHO describe quality of care as ‘the degree to which health services for individuals and populations increase the likelihood of desired health outcomes and are consistent with current professional knowledge.’^1^ ^(p12)^ Quality is an outcome of performance. A study conducted by Beadle‐Brown *et al.* (2008) in England highlighted that standards need to capture service‐users’ experiences and outcomes in order to have quality assurance.^47^ This study found that measures of service user outcomes did not correlate with related processes within the National Minimum Standards for Care Homes for Younger Adults (2002) and subsequently affected credibility and effectiveness in inspection programmes.^47^ This was further supported by Cunningham *et al.* (2020) who wrote a review in the context of Northern Ireland and examined how standards and quality measures are used to regulate health and social care services. Findings suggested that standards would be more effective if based on measurable lived experiences from service‐users and care outcomes.^43^ However, it is noteworthy that the term ‘outcome’ is not used in the definitions of standards extracted from standard‐setting bodies in England and Northern Ireland.

Perform or performance was used in five definitions^4^, ^6^, ^23^, ^24^, ^26^ selected from the search but performance in itself is made up of many concepts including efficiency, effectiveness, quality, safety and human resources.^48^ A study summarising indicator‐words relative to performance in hospitals using a co‐word analysis yielded 2070 articles for inclusion dating from 1975 to 2014.^48^ In this analysis the most frequent words used with performance in hospitals were ‘mortality’ and ‘efficiency’ followed by ‘quality of care’, ‘quality improvement’, ‘discharge’, ‘length of stay’ and ‘clinical outcome.’^48^ The findings from this study reflect the pioneering work of Dr Renest Codman on patient outcomes management in the USA. This involved the practice of tracking the patient's journey in relation to mortality, harm, and length of hospital stay to reflect overall performances in patient safety.^49^ Interestingly, the IOM uses performance three times in their definition and describe performance as acceptable or excellent. The WHO report that quality needs to be monitored and assessed to promote improvements.^1^ Consequently, performance must be measured to ascertain its effects and efficiencies which may explain the use of ‘measure/measurable’ in four definitions retrieved in this study.^8^, ^15^, ^24^, ^27^

Variations in describing and interpreting health and social care standards may depend on the standard‐setting bodies' overall structures and behaviours. An analysis of the organisational structures was not conducted as part of this review. However, some organisations could be distinguished by their oversight processes, such as accreditation, regulation or inspection requirements. For example, the quality standards for health and social care in Northern Ireland reflect the minimum level of practice required to achieve safe and effective care. These standards are used as a benchmark for quality assessment, both by local care services and the Northern Ireland Regulatory Body.^20^ This is also reflected in their healthcare legislation where standards are referred to as ‘statements of minimum standards.’ However, the term ‘statement’ does not feature in their definition of standards. The Australian Health Service Safety and Quality Standards and the Danish Healthcare Quality Standards set out the requirements for accreditation assessments.^4^, ^27^ Definitions used by these organisations describe standards as a process^4^ that is measured^27^ which captures the evaluation element that is associated with accreditation in healthcare.

In addition, national legislative enactments may also play a role in describing what is meant by standards in health systems. For example, section ten of the National Health Services Scotland Act 1978 uses the term ‘standards’ and ‘outcomes’ consecutively and the definition of standards used by the Scottish government includes the term ‘outcomes’.^18^, ^31^ Similarly, part eight of the Health and Social Care Act 2012 enacted in England uses the term ‘a quality standard’ and ‘statement of standards’,^29^ which may explain the inclusion of ‘quality’ and ‘statement’ in the definition of standards used by NICE.^8^ In addition, this legislation refers to a statement of standards as a document that includes advice to the Secretary of State regarding the quality of providing health care. The definition of standards used by the Welsh government places emphasis on level of quality, and section nine of the Social Services and Well‐being (Wales) Act 2014 refers to standards as ‘quality standards’ to be achieved in the provision of care and support.^32^ Section ten of the Health Act 2007 in Ireland states that standards are those set by the authority, which is the Irish standard‐setting body, HIQA.^36^ They are based on safety and quality of services which may explain why the terms ‘quality’ and ‘safety’ feature in the definition of health and social care standards used by HIQA. Interestingly, while the Health and Disability Services Act 2001 in New Zealand does not provide an explicit definition of standards, Section 21 of the Act sets out what the standards should include, for example, general statements of appropriate care delivery outcomes for providers of health or disability services, statements of outcomes for aspects of providing health or disability services.^35^ In addition, such statements may include means of achieving the outcomes and/or criteria for assessing whether the outcomes are appropriate. The interpretation section of the legislation states that the terms ‘service standards’ means being approved by the Minister. Similarly in Wales, section nine of the Social Services and Well‐being (Wales) Act 2014 differentiates quality standards between categories of care and support and categories of people who need care and support.^32^ However, it is important to note that standards by themselves do not form part of primary legislation. They are embodied in the organisation that has been established under legislation with responsibility to develop health and social care standards. In addition, they may be embodied in a legislative regulatory or licencing framework and thus may explain why explicit definitions of standards were not retrieved from searches of Health Acts (or equivalent) internationally.

The WHO recommend the use of acceptable local language to facilitate a shared understanding when defining quality.^1^ Developing a definition of standards that incorporates ‘acceptable local language’ may prove difficult if trying to factor the multiple concepts of standards into one single definition. This may in part, explain the lack of a unified definition for standards. However, the quest for quality is similar globally.^1^ Shared health system goals encompass reducing harm to patients, improving clinical effectiveness, building system capacity, person centredness and governance strengthening and accountability.^1^ Ironically, countries have shared visions but not a shared language when defining quality improvement interventions like standards.

### 4.1 Implications for practice

In general, the absence of a universal definition for any construct can act as a barrier for researchers conducting comprehensive searches of the literature on any topic. Standards are broad and are made up of many varied components. Researchers and reviewers will need to be cognisant of this complex variation when synthesising literature relating to health and social care standards. In addition, comparing standards between standard‐setting bodies should be undertaken with caution. Standards may be set in the context of the legislation that underpins the health and social care system in which they are developed and subsequently implemented, hence, countries using standards that have been sourced from other countries need to adapt them to ensure that they are applicable in the context of another health and social care system. Developing a universal definition will prove difficult to include all the elements of what standards are and, given that any definition of standards is only meaningful in the context to which it applies. Moreover, the variation in definitions of standards as identified in this review is justifiable and necessary given the different health and social care systems internationally.

### 4.2 Strengths and limitations

A strength of this study is that the search strategy was informed by subject matter experts who had conducted a review of methodologies for developing national standards and guidance for health and social care services used by organisations internationally. This study included a search of legislation, internationally, taking into account the foundation of standard‐setting bodies and standards in health and social care systems. The search was limited to 12 standard‐setting bodies. Two standard‐setting bodies have international remit in setting standards and ten standard‐setting bodies have similar roles and responsibilities to each other. This may have resulted in a narrow focus. A broader search may have yielded organisations with other definitions of standards or definitions weighted differently towards terms used, given that they likely operate in other types of health and social care systems. However, it is unlikely that this would have affected the findings from this review in that a main finding is that variation exists between definitions of standards. The changing nature of websites means that addresses, domains and content are not static. This limits the reproducibility of the exact search and returns included in this review. Nonetheless, this narrative review is informative in that it has identified the similarities and differences in definitions of standards and has highlighted the complexities and contextual factors associated with standards at a recent, fixed point in time.

## CONCLUSION

Legislation does not provide an explicit definition of standards for health and social care despite government parties, internationally enacting the establishment of standard‐setting bodies for health and social care into law. Definitions of standards used by standard‐setting bodies have some commonalities such as being quality statements, measurable and related to performances. The less commonly used descriptors include a process, set (of), evidence‐base, outcome, safe, and guidance. Health and social care standards set by standard‐setting bodies used in this review are nationally endorsed standards. As such, standards that are developed and implemented are determined by a country's health and social care system. Researchers, standards developers and policy‐makers are encouraged to exercise caution when comparing standards between countries. Alongside evaluating the set of standards for an area or service, there is a need to identify the underpinning definition of standards used by the standard‐setting body and understand the variations that exist between the countries for comparison. Using an evidence base is fundamental to the standards development processes. This perhaps suggests a need to make those attributes that are central to health and social care standards more explicit within underpinning definitions, thus facilitating a deeper understanding of what standards are and promoting credibility with their adoption in practice. In addition, this research may serve as a starting point for standard‐setting bodies to reflect on the terms used to define their standards and reconstruct terminology to represent the nature of standards and the standards development process.

## CONFLICTS OF INTEREST

The author, Yvonne Kelly is undertaking a PhD studentship in HIQA and authors, Niamh O’Rourke, Rachel Flynn and Laura O’Connor are currently employed by HIQA, a national organisation with the responsibility of developing health and social care standards for Ireland.

## ETHICS STATEMENT

Not applicable.

## Supporting information

Supporting Information S1

Supporting Information S2

## Data Availability

The data that supports the findings of this study are available in the supplementary material of this article.

## References

[1] OECD, WHO, World Bank Group . Delivering Quality Health Services: A Global Imperative; 2018. Accessed 16 August 2020. 10.1787/9789264300309-en

[2] International Organization for Standardization (ISO) . Standards. Accessed 11 December 2020. https://www.iso.org/home.html

[3] Oxford English Dictionary . Standards. Oxford University Press; 2022. https://www.oed.com/

[4] Australian Commission on Safety and Quality in Health Care . National Safety and Quality Health Service Standards 2017; 2017:1‐75. Accessed 29 January 2021. https://www.safetyandquality.gov.au/sites/default/files/2019‐04/National‐Safety‐and‐Quality‐Health‐Service‐Standards‐second‐edition.pdf

[5] Australian Commission on Safety and Quality in Health Care . Creating Safer, Better Health Care, the Impact of the National Safety and Quality Health Service Standards; 2018:1‐11. Accessed 01 February 2021. https://www.safetyandquality.gov.au/sites/default/files/migrated/NSQHS‐Impact‐Report.pdf

[6] Institute of Medicine (US) . Committee on quality of health care in America. Setting performance standards and expectations for patient safety. In: Kohn LT , Corrigan JM , Donaldson MS , eds. To Err is Human: Building a Safer Health System. National Academies Press (US); 2000:132‐152. 25077248

[7] Madden D , Organisational and Professional Regulatory Framework . Building a Culture of Patient Safety: Report of the Commission on Patient Safety and Assurance. Department of Health; 2008:105‐120. Accessed August 15, 2020. https://www.gov.ie/en/publication/5d9570‐building‐a‐culture‐of‐patient‐safety‐report‐of‐the‐commission‐on‐pat/

[8] National Institute for Health and Care Excellence (NICE) . The NICE Quality Standards Programme; 2016. Accessed 18 August 2020. https://www.nice.org.uk/

[9] Aged Care Quality and Safety Commission, Regulatory Strategy 2020. Australian Government. 2020. Accessed January 10, 2021. https://www.agedcarequality.gov.au/resources/regulatory‐strategy

[10] Ministry of Health, . Certification of health care services. 2020. Accessed January 10, 2021. https://www.health.govt.nz/our‐work/regulation‐health‐and‐disability‐system/certification‐health‐care‐services

[11] Health Information and Quality Authority . Assessment Judgment Framework for Designated Centres for Older People. Regulation of Health and Social Care Services; 2018. Accessed January 11, 2021. https://www.hiqa.ie/sites/default/files/2018‐02/Assessment‐Judgment‐Framework‐DCOP_Guidance.pdf

[12] Regulation and Quality Improvement Authority . Provider Guidance 2019‐20, Domiciliary Care Agencies; 2019. Accessed 10 January 2021. https://www.rqia.org.uk/

[13] Care Quality Commission . Regulations for Service Providers and Managers; 2017. Accessed 11 January 2021. https://www.cqc.org.uk

[14] Introduction WHO . Frequently asked questions. In: Groene O , ed. Implementing health promotion in hospitals: Manual and self‐assessment forms. WHO Regional Office for Europe; 2006:7‐9. Accessed August 18, 2020. https://apps.who.int/iris/handle/10665/107737

[15] Health Quality Ontario . Quality Standards Process and Methods Guide; 2017. Accessed 18 August 2020. https://www.hqontario.ca/Portals/0/documents/evidence/Quality_Standards_Process_andMethods_Guide‐‐Oct_2017.pdf

[16] Health Information and Quality Authority (HIQA) . National Standards for Safer Better Healthcare; 2012. Accessed 12 Novemeber 2020. https://www.hiqa.ie/sites/default/files/2017‐01/Safer‐Better‐Healthcare‐Standards.pdf

[17] Standards New Zealand . Health and Disability Services. Ministry of Health. 2008:13. Accessed January 29, 2021. https://www.standards.govt.nz/

[18] Health and Social Care Standards My Support, My Life. Scottish Government. 2017. Accessed August 19, 2020. https://www.gov.scot/publications/health‐social‐care‐standards‐support‐life/

[19] Fletcher D , Sarkar M . Psychological resilience: a review and critique of definitions, concepts, and theory. Eur Psychol. 2013;18(1):12‐23. 10.1027/1016-9040/a00012

[20] HIQA . International Review of the Methodologies for Developing National Standards and Guidance for Health and Social Care Services; 2018. Accessed 29 January 2021. https://www.hiqa.ie/

[21] The National Board of Health and Welfare . Socialstyrelsen; 2015. Accessed 17 August 2020. https://www.socialstyrelsen.se/

[22] National Standards for Disability Services . Department of Social Services. Government; 2013. Accessed August 18, 2020. https://www.dss.gov.au/sites/default/files/documents/06_2015/nsds_full_version.pdf

[23] Health and Care Standards . Welsh Assembly Government; 2015. Accessed 12 November 2020. http://www.wales.nhs.uk/sitesplus/documents/1064/24729_Health%20Standards%20Framework_2015_E1.pdf

[24] The Quality Standards for Health and Social Care. Department of Health, Social Services and Public Safety. 2006. Accessed January 11, 2021. https://www.health‐ni.gov.uk/

[25] The Standard Programme, Key Concepts . The Standard Programme, Key Concepts, Centre for Quality Improvement. Danish Quality Model for Social Services; 2017. Accessed 18 August 2020. https://www.socialkvalitetsmodel.dk/Standardprogrammet/centrale‐begreber/

[26] Why Standards? an Introduction to the Benefits of Standards. , Ministry of Business, Innovation, Employment. 2017. Accessed January 11, 2021. https://www.Standards.govt.nz/assets/About‐us/Resources/Why‐Standards‐brochure‐2016‐v4‐Web.pdf

[27] Accreditation Standards for hospitals . IKAS Danish Institute for Quality and Accreditation in Healthcare. 2nd version; 2016. Accessed 18 August 2020. https://www.ikas.dk/den‐danske‐kvalitetsmodel/ddkm‐in‐english/accreditation‐Standards/

[28] Australian Government . National Health Reform Act 2011, Chapter 2. Accessed 09 January 2021. https://www.legislation.gov.au/Details/C2016C01050

[29] United Kingdom, England . Health and Social Care Act 2012, Section 8. Accessed 09 January 2021. https://www.legislation.gov.uk/ukpga/2012/7/part/8/enacted

[30] Law O . The Excellent Care for All Act (ECFAA) 2010. Accessed 09 January 2021. https://www.ontario.ca/laws/statute/10e14%23BK1

[31] Scottish Government . National Health Services Scotland Act 1978, Section 10H. Accessed 09 January 2021. https://www.legislation.gov.uk/ukpga/1978/29/contents

[32] National Assembly for Wales . Social Services and Well‐Being (Wales) Act 2014, Section 9. Accessed 09 January 2021. https://www.legislation.gov.uk/anaw/2014/4/section/9

[33] Northern Ireland Orders in Council . The Health and Personal Social Services (Quality, Improvement and Regulation) (Northern Ireland) Order; 2003. Accessed 09 January 2021. https://www.legislation.gov.uk/nisi/2003/431/article/38/made

[34] Danish Ministry of Health and the Elderly . Health Act 2010. Accessed January 09, 2021. https://www.retsinformation.dk/eli/lta/2010/913%23K77

[35] New Zealand Government . Health and Disability Services (Safety) Act 2001;Part 1:2 2. Accessed February 05, 2021. https://www.legislation.govt.nz/act/public/2001/0093/latest/DLM120553.html

[36] Government of Ireland . Health Act 2007, Section 8. Accessed January 10, 2021. http://www.irishstatutebook.ie/eli/2007/act/23/section/10/enacted/en/html%23sec10

[37] Danish Ministry of Social Affairs and the Interior . Consolidation Act on Social Services 2015. Accessed January 10, 2021. http://english.sm.dk/media/14900/consolidation‐act‐on‐social‐services.pdf

[38] Braun V , Clarke V . Using thematic analysis in psychology. Qual Res Psychol. 2006;3(2):77‐101. 10.1191/1478088706qp063oa

[39] Campbell M . Getting to grips with statistics: understanding variables. Br J Midwifery. 2016;24(10):738‐741. 10.12968/bjom.2016.24.10.738

[40] Oxford English Dictionary . Oxford English Dictionary. Statements” Oxford University Press; 2020. Accessed August 20, 2020. https://www.lexico.com/definition/statement

[41] NAM . Strategic Plan 2018‐2023, Goalposts for a Healthier Future. 2018. Accessed 19 July 2020. https://nam.edu/wp‐content/uploads/2017/10/National‐Academy‐of‐Medicine‐2018‐2023‐Strategic‐Plan.pdf

[42] Kinney ED . The origins and promise of medical standards of care. AMA J Ethics. 2004;6(12):574‐576. 10.1001/virtualmentor.2004.6.12.mhst1-0412 23260291

[43] Cunningham S , Taylor BJ , Murphy A . Standards in regulating quality of adult community health and social care: systematic narrative review. J Evid Base Soc Work. 2020;17(4):457‐468. 10.1080/26408066.2020.1770647 32498667

[44] Reeves CA , Bednar DA . Defining quality: alternatives and implications. Acad Manag Rev. 1994;19(3):419‐445. 10.5465/amr.1994.9412271805

[45] Oxford English Dictionary . Quality. Oxford University Press; 2020. Accessed August 20, 2020. https://www.lexico.com/definition/quality

[46] Cambridge English Dictionary . Quality 2020. Accessed 20 August 2020. https://dictionary.cambridge.org/

[47] Beadle‐Brown J , Hutchinson A , Mansell J . Care standards in homes for people with intellectual disabilities. J Appl Res Intellect Disabil. 2008;21(3):210‐218. 10.1111/j.1468-3148.2007.00400.x

[48] Markazi‐Moghaddam N , Mohammad A , Ravaghi H , Rashidian A , Khatibi T , Jame SZB . A knowledge map for hospital performance concept: extraction and analysis: a narrative review article. Iran J Public Health. 2016;45(7):843. 27516990 PMC4980338

[49] Brand RA . Biographical sketch: ernest amory codman, MD (1869–1940). Clin Orthop Relat Res. 2013;471(6):1775‐1777. 10.1007/s11999-012-2750-40 23247819 PMC3706647

