# Supplementary material for: Definitions of health and social care standards used internationally: A narrative review
Source: Int J Health Plann Manage. 2022 Sep 20;38(1):40–52. doi: 10.1002/hpm.3573 (PMC10087784; doi:10.1002/hpm.3573)
Supplement: Supplementary file 1 — Supporting Information S1 [file HPM-38-40-s002.docx]

**Supplementary File 1- List of standard-setting bodies, Health Acts and websites consulted to extract a definition of standards**

**1a. List of Websites consulted to extract a definition of standards**

|  | **Country** | **Website Name: Standards-setting Body** | **Website Link** |
| --- | --- | --- | --- |
|  | Australia | Australian Commission on Safety and Quality in Health Care | <https://www.safetyandquality.gov.au/> |
|  | Australia | Australian Governemnt, Department of Social Services | <https://www.dss.gov.au/> |
|  | Denmark | IKAS Danish Institute for Quality and Accreditation in Healthcare | <https://www.ikas.dk/forside/> |
|  | Denmark | Danish quality model in the social area | <https://www.socialkvalitetsmodel.dk/> |
|  | England | National Institute for Health and Care Excellence | <https://www.nice.org.uk/> |
|  | England | Social Care Institute for Excellence (SCIE) | <https://www.scie.org.uk/> |
|  | Ireland | Health Information and Quality Authority | <https://www.hiqa.ie/> |
|  | New Zealand | Standards New Zealand (Ministry of Business, Innovation and Employment) | [https://www.Standards.govt.nz/](https://www.standards.govt.nz/) |
|  | Northern Ireland | Department of Health, Social Services and Public Safety | <https://www.health-ni.gov.uk/> |
|  | Ontario | Health Quality Ontario | <https://www.hqontario.ca/> |
|  | Scotland | Scottish Government | <https://www.gov.scot/> |
|  | Sweden | Socialstyrelsen (The Swedish National Board of Health and Welfare) | <https://www.socialstyrelsen.se/en/> |
|  | Wales | Welsh Assembly Government | <https://gov.wales/> |
|  | United States of America | Institute of Medicine | <https://www.ncbi.nlm.nih.gov/books/NBK225181/> |
|  | WHO | WHO | <https://www.euro.who.int/> |

**1b. List of Health Acts consulted to extract a definition of standards**

|  | **Country** | **Title of Health Act** | **Website Link** |
| --- | --- | --- | --- |
| 1. | Australia | National Health Reform Act 2011 | <https://www.legislation.gov.au/Details/C2016C01050> |
| 2. | Denmark | Health Act 2010 | <https://www.retsinformation.dk/eli/lta/2010/913#K77> |
| 3. | Denmark | Consolidation Act on Social Services 2015 | <http://english.sm.dk/media/14900/consolidation-act-on-social-services.pdf> |
| 4. | England | Health and Social Care Act 2012 | <https://www.legislation.gov.uk/ukpga/2012/7/part/8/enacted> |
| 5. | Ireland | Health Act 2007 | <http://www.irishstatutebook.ie/eli/2007/act/23/section/10/enacted/en/html#sec10> |
| 6. | New Zealand | Health and Disability Services (Safety) Act 2001 | <https://www.legislation.govt.nz/act/public/2001/0093/latest/DLM120553.html> |
| 7. | Northern Ireland | The Health and Personal Social Services (Quality, Improvement and Regulation) (Northern Ireland) Order 2003 | <https://www.legislation.gov.uk/nisi/2003/431/article/38/made> |
| 8. | Ontario | The Excellent Care for All Act 2010 | <https://www.ontario.ca/laws/statute/10e14#BK1> |
| 9. | Scotland | National Health Services Scotland Act 1978 | <https://www.legislation.gov.uk/ukpga/1978/29/contents> |
| 10. | Wales | Social Services and Well-being (Wales) Act 2014 | <https://www.legislation.gov.uk/anaw/2014/4/section/9> |
