# Supplementary material for: Definitions of health and social care standards used internationally: A narrative review
Source: Int J Health Plann Manage. 2022 Sep 20;38(1):40–52. doi: 10.1002/hpm.3573 (PMC10087784; doi:10.1002/hpm.3573)
Supplement: Supplementary file 2 — Supporting Information S2 [file HPM-38-40-s001.docx]

**Supplementary File 2-Search Strategy: Grey Literature**

| **Australia: Australian Commission on Safety and Quality in Health Care** |
| --- |
| <https://www.safetyandquality.gov.au/>  Screen Headings  “Publications and Resources”  Subheadings  “Popular publications and resources”  n=6  Link:  NSQHS Standards resources  n=16  NSQHS Standards 2^nd^ Edition (2017) |
| **Australia: Australian Governemnt, Department of Social Services** |
| <https://www.dss.gov.au/>  Screen Headings  Search Function: “Standards”  n=1062  Filter: ‘last updated’  Titles screen on first page  n=40  National Standards for Disability Services (2013)  <https://www.dss.gov.au/sites/default/files/documents/06_2015/nsds_full_version.pdf> |
| **Denmark: IKAS Danish Institute for Quality and Accreditation in Healthcare** |
| <https://www.ikas.dk/forside/>  English version  Screen Headings  Accreditation Standards  Resources  n=3 (current accreditation Standards available in English)  n=2 (most recently published)  Excluded n=1 (professional Standards)  No definition retrieved from  “Accreditation Standards for community pharmacies, 3nd version, 2018”  Re-read homepage and definition extracted from text on homepage |
| **Denmark: Danish quality model in the social area** |
| <https://www.socialkvalitetsmodel.dk/>  English version  Screen Headings  Standards Programme  Sub-heading on Key concepts with link to find all Standards together  Download Standards Booklet |
| **UK: National Institute for Health and Care Excellence** |
| <https://www.nice.org.uk/>  Screen Headings  “Standards & Indicators”  “Quality Standards”  n= 193  titles screened (n=193)  Topic Specific Standards  “Process Guide – how to develop the Standards” |
| **Ireland: Health Information and Quality Authority** |
| <https://www.hiqa.ie/>  Screen Headings  Standards & Quality  “National Standards and Guidance”  n=17  Titles screened  Overarching Standards-“National Standards for Safer Better Healthcare” |
| **New Zealand: Ministry of Business, Innovation and Employment** |
| [https://www.Standards.govt.nz/](https://www.standards.govt.nz/)  Screen Headings  Search function: “healthcare Standards”  n=14, 753  1^st^ 20 titles screened: irrelevant returns  “social care Standards”  n= 15, 282  1^st^ 20 titles screened: irrelevant returns  Link: Resources  n=6  “Why Standards? An introduction to the benefits of Standards” |
| **Northern Ireland: Department of Health, Social Services and Public Safety** |
| <https://www.health-ni.gov.uk/>  Screen Headings  “Safety & Quality Standards”  Subheading: Standards for safety and quality  “Quality Standards for health and social care” |
| **WHO** |
| <https://www.euro.who.int/>  Screen Headings  Search function: “healthcare Standards”  n= 822  1^st^ 50 titles screened  Search function: “social care Standards”  n= 33  Titles screened  “Health promotion in hospitals” |
| **Scotland: Scottish Government** |
| <https://www.gov.scot/>  Screen Headings  search function: “healthcare Standards”  n= 82  titles screened  “Health & Social care Standards: my support, my life” 2017 |
| **Wales: Welsh Assembly Government** |
| <https://gov.wales/>  Screen Headings  Search function: “healthcare Standards”  n=7320  1^st^ 20 titles screened  “health and social care Standards 2015” |
| **Sweden: Socialstyrelsen (National Board of Health and Welfare)** |
| <https://www.socialstyrelsen.se/en/>  Screen Headings  Search function: “healthcare Standards”  Excluded |
| **Ontario: Health Quality Ontario** |
| <https://www.hqontario.ca/>  Screen Headings  “Evidence to improve care”  Quality Standards  Link: About Quality Standards  “Process and Method Guide” |
